# Prediction of immunotherapy response of bladder cancer with a pyroptosis-related signature indicating tumor immune microenvironment

**DOI:** 10.3389/fphar.2024.1387647

**Published:** 2024-06-25

**Authors:** Zihan Xu, Yujie Zhao, Yong Zhang, Xiaowei Liu, Linlin Song, Meixu Chen, Guixiu Xiao, Xuelei Ma, Hubing Shi

**Affiliations:** ^1^ Institute for Breast Health Medicine, State Key Laboratory of Biotherapy, West China Hospital, Sichuan University and Collaborative Innovation Center, Chengdu, Sichuan, China; ^2^ Department of Medical Oncology, Cancer Center, West China Hospital, Sichuan University, Chengdu, Sichuan, China; ^3^ Department of Biotherapy, West China Hospital and State Key Laboratory of Biotherapy, Sichuan University, Chengdu, Sichuan, China

**Keywords:** pyroptosis, bladder cancer, tumor microenvironment, immunotherapy, predictive model

## Abstract

**Background:**

Although prognostic models based on pyroptosis-related genes (PRGs) have been constructed in bladder cancer (BLCA), the comprehensive impact of these genes on tumor microenvironment (TME) and immunotherapeutic response has yet to be investigated.

**Methods:**

Based on expression profiles of 52 PRGs, we utilized the unsupervised clustering algorithm to identify PRGs subtypes and ssGSEA to quantify immune cells and hallmark pathways. Moreover, we screened feature genes of distinct PRGs subtypes and validated the associations with immune infiltrations in tissue using the multiplex immunofluorescence. Univariate, LASSO, and multivariate Cox regression analyses were employed to construct the scoring scheme.

**Results:**

Four PRGs clusters were identified, samples in cluster C1 were infiltrated with more immune cells than those in others, implying a favorable response to immunotherapy. While the cluster C2, which shows an extremely low level of most immune cells, do not respond to immunotherapy. CXCL9/CXCL10 and SPINK1/DHSR2 were identified as feature genes of cluster C1 and C2, and the specimen with high CXCL9/CXCL10 was characterized by more CD8 + T cells, macrophages and less Tregs. Based on differentially expressed genes (DEGs) among PRGs subtypes, a predictive model (termed as PRGs score) including five genes (CACNA1D, PTK2B, APOL6, CDK6, ANXA2) was built. Survival probability of patients with low-PRGs score was significantly higher than those with high-PRGs score. Moreover, patients with low-PRGs score were more likely to benefit from anti-PD1/PD-L1 regimens.

**Conclusion:**

PRGs are closely associated with TME and oncogenic pathways. PRGs score is a promising indicator for predicting clinical outcome and immunotherapy response.

## 1 Introduction

Bladder cancer (BLCA) has been reported as the 11th most prevalent cancer globally, with about 550,000 new cases per annum (Lenis et al., 2020; Teoh et al., 2020). According to epidemiological investigations, smoking is the most crucial risk factor for BLCA. Furthermore, strategies to inhibit smoking have indicated improved survival of patients with lung cancer; however, it has not indicated successful outcomes in BLCA patients (Cumberbatch et al., 2016). These observations suggest that BLCA has unique genetic/epigenetic alterations, and immune responses (Cao et al., 2020). Based on the pathological characteristics, BLCA can be divided into non-muscular invasive and muscular invasive types (Wang et al., 2023). However, different BLCA has different challenges, for instance, non-muscular invasive BLCA has a high recurrence rate after surgery, while muscular invasive BLCA indicates a very poor prognosis, with only few patients surviving more than 5 years. (Chou et al., 2016; Ghandour et al., 2019). In recent years, immunotherapy has made great progress. Anti-programmed cell death protein ligand-1 antibody (αPD-L1) has been approved by the US Food and Drug Administration (FDA) for BLCA treatment since 2016, with its usage spanning from non-muscle invasive to metastatic disease (Schneider et al., 2019). However, a significant number of BLCA patients do not respond to these treatments (Galsky et al., 2020; Powles et al., 2021). On the one hand, numerous tumors exhibit an “immune-cold” phenotype, characterized by an immunosuppressive tumor microenvironment (TME), rendering them unresponsive to current immunotherapeutic agents (Lee et al., 2022; Zhang et al., 2023). On the other hand, αPD-L1 is a viable choice only for programmed cell death protein-ligand 1 (PD-L1) positive BLCA patients, while PD-L1 expression varies between individuals (Afonso et al., 2020). Therefore, it is essential to identify driving factors in genetic/epigenetic and immune level and construct a new predictive model for immunotherapy response and survival in BLCA (He et al., 2021).

Pyroptosis is a kind of programmed cell death, with inflammation triggered by detrimental signals or pathogenic microbial infection (Frank and Vince, 2019). Furthermore, it is manifested with cell swelling, lysis, and cytoplasmic content secretion. It is an essential host resistance mechanism against infection by pathogenic microbes. However, increased or uncontrolled pyroptosis is harmful and even fatal for the host. Previous studies indicated that pyroptosis was linked with the initiation and progression of various cancers, as well as affecting the TME. Much literature has revealed that pyroptosis is critically involved in tumor development (Fang et al., 2020). Additionally, crosstalk between TME and pyroptosis has also been indicated (Orning et al., 2019; Erkes et al., 2020). TME primarily comprises endothelial cells, fibroblasts, extracellular matrix, immune and inflammatory cells, and diffuse chemokines and cytokines, which are notably associated with tumor initiation and progression (Runa et al., 2017). Currently, because of technical limitations, most research only investigated 1 or 2 pyroptosis-related genes (PRGs) in cell and animal models. However, antitumor effects require highly coordinated interactions among many genes. Therefore, comprehensive research on the characteristics of various PRGs-mediated TME cell infiltration is essential and may furnish crucial data on mechanisms of BLCA oncogenesis and progression, as well as predict the immunotherapy response.

This study aims to classify subtypes of different immune infiltrates by analyzing the PRGs in BLCA patients and construct a scoring model, for prognosis prediction and clinical treatment guidance. TCGA-BLCA patients were used to identify and validate four pyroptosis-linked subtypes that were related to immune infiltration and prognosis. Based on differentially expressed genes (DEGs) assessed by the 4 pyroptosis subtypes, the patients were categorized into two geneClusters. Moreover, the LASSO-Cox method was employed to establish the pyroptosis correlation model and elucidate the risk score. Overall, the four pyroptosis-related subtypes and scoring systems constructed in this study could predict immune infiltration, prognosis, and immunotherapy response. Additionally, the acquired data indicated a potential link between TME, pyroptosis, immunotherapy response, and prognosis in BLCA patients.

## 2 Materials and methods

### 2.1 Data sources

**FIGURE 1:**
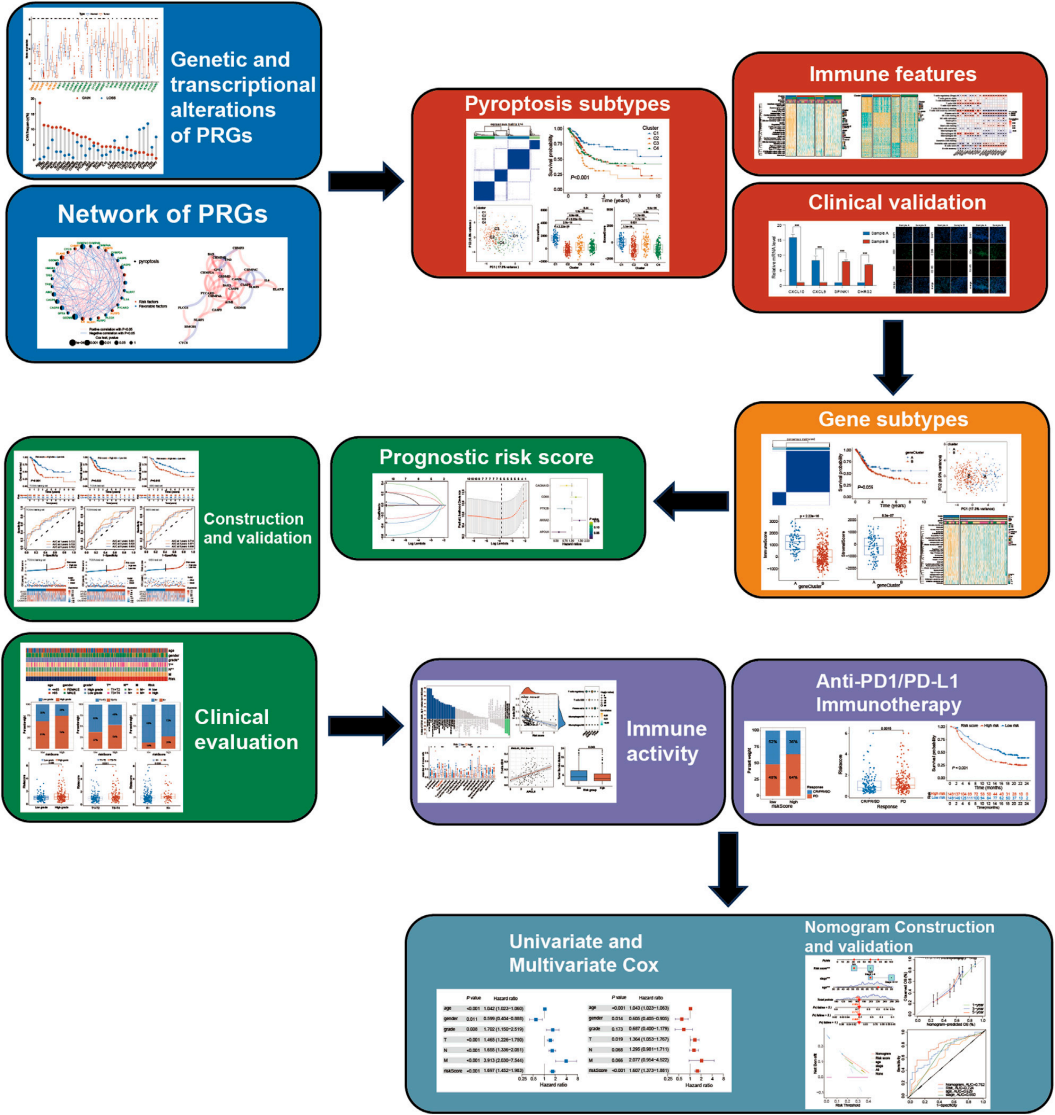
Study flow chart.

Figure 1 indicates the study’s flowchart. BLCA sample’s clinicopathological and gene expression (fragments per kilobase million, FPKM) data were acquired from The Cancer Genome Atlas (TCGA; 406 BLCA patients) (https://portal.gdc.cancer.gov/) and the Gene Expression Omnibus (GEO; 165 BLCA patients) (https://www.ncbi.nlm.nih.gov/geo/). Detailed information on the selected BLCA patients is given in Supplementary Table S1. Clinical information included tumor grade, age, TNM stage, follow-up time, sex, and survival status. Data in this research were downloaded from publicly available datasets, therefore ethics committee approval was not required.

### 2.2 Consensus clustering for pyroptosis-related genes in BLCA

Using the “REACTOME_PYROPTOSIS” item of MSigDB (http://www.broad.mit.edu/gsea/msigdb/) and previous literature (Ye et al., 2021), 52 PRGs were identified (details in Supplementary Table S2). Furthermore, based on the expression profiles of these PRGs, unsupervised clustering was conducted by using the “ConsensusClusterPlus” package to categorize participants into distinct molecular subtypes (termed: PRGs clusters). To ensure the cluster’s reliability, clustering was repeated 1,000 times. DEGs between the different PRGs clusters were identified using the “limma” package in R with a fold-change of 1.5 and an adjusted *p*-value of <0.001. Finally, we screened and identified 240 DEGs. Additionally, based on expression of DEGs, unsupervised clustering was carried out to classify patients into distinct clusters (termed: geneClusters).

### 2.3 Construction and validation of the PRGs-DEGs risk score system

Using the “caret” package, TCGA acquired 406 BLCA patients who were randomly categorized in a 1:1 ratio into the train and test cohorts. Then the risk scoring system was constructed in the TCGA-train cohort. Briefly, survival-related genes were assessed via the univariate Cox regression using PRGs-DEGs, and then LASSO regression was carried out to exclude overfitting genes. To build the predictive model, the filtered genes were subjected to multivariate Cox regression using the forward/backward method.

The PRGs-DEGs risk score was calculated as follows:

$$\text{PRGs}-\text{DEGs risk score}=\Sigma \;\left(\text{Expi }*\text{ coefi}\right)$$

Where Expi and Coefi indicated the expression of each gene and risk coefficient, respectively. According to the median risk score value from the TCGA-train cohort, other patients from the TCGA-test and GEO cohorts were categorized into high-risk (HR) and low-risk (LR) subgroups. The log-rank test and the Kaplan-Meier curve were applied to determine the survival differences between LR and HR subgroups. Moreover, the area under the curve (AUC) value of the receiver operating characteristic (ROC) curve was assessed to elucidate the reliability of the predictive model.

### 2.4 Clinical correlation and stratification analyses

The PRGs cluster’s clinical value was identified via consensus clustering. Moreover, the association of PRGs clusters with clinicopathological features (including tumor grade, age, TNM stage, and sex) was compared. Furthermore, Kaplan-Meier curves were drawn by the “survival,” whereas using the “survminer” R packages, the differences between overall survival (OS) and progression-free survival (PFS) among different PRGs clusters were assessed. Additionally, Chi-square tests were utilized to elucidate the relationship between PRGs-DEGs risk score and clinical features. Furthermore, univariate and multivariate analyses were conducted to evaluate if the risk score was independent of other clinicopathological parameters using the BLCA cohort. A stratified analysis was also carried out to assess if the risk score maintained its predictive ability across subgroups based on the aforementioned clinicopathological parameters.

### 2.5 Tumor purity analysis and single sample gene set enrichment analysis (ssGSEA)

According to the gene expression profiles, the ESTIMATE algorithm in R was employed to calculate the tumor purity of each patient including immune and stromal scores. Furthermore, the expression profiles were converted into the scoring matrix of hallmark pathways/phenotypes or immune infiltrations via the R “GSVA” package (method = “ssGSEA”). The reference hallmark gene set was acquired from GSEA (https://www.gsea-msigdb.org/gsea). Then, differential analyses of ssGSEA scores were carried out for distinct PRGs clusters, geneClusters, or HR/LR score clusters. As a continuous variable, the PRGs-DEGs risk score was evaluated for the correlation with ssGSEA scores related to hallmark pathways/phenotypes and immune infiltrations. Additionally, the association of the risk signature gene levels with ssGSEA scores of substantially altered pathways/phenotypes and immune infiltrations was assessed, respectively.

### 2.6 Quantitative real-time PCR (qRT-PCR)

The BLCA and para-carcinoma tissues used in this study were gifted from another research group. This research was authorized by the Institutional Research Ethics Committee of West China Hospital. For the detection of the mRNA levels of marker genes and prognostic genes, qRT-PCR was employed. Briefly, whole RNA was acquired using Trizol (Thermo Fisher Scientific), reverse transcribed to single-strand cDNA. For qRT-PCR amplification, qRT-PCR was conducted using SYBR^®^ Green Real-time PCR Master Mix (TOYOBO). GAPDH was utilized for normalizing the relative mRNA levels. Supplementary Table S3 enlists the sequences of primers employed.

### 2.7 Immunofluorescence (IF)

The tissues of BLCA and para-carcinoma obtained above were paraffin-embedded. CD163, CD8, and FoxP3 were used as specific markers for tumor-associated macrophages (TAMs), CD8 + T cells, and regulatory T cells (Tregs), respectively. The samples were dewaxed in xylene, rehydrated using alcohol, blocked with the help of endogenous peroxidase, treated overnight with specific antibodies at 4°C in a humidified box, and then tagged with secondary antibodies. Lastly, the samples were counterstained with hematoxylin and visualized by diaminobenzidine.

### 2.8 Immunotherapy susceptibility analysis

Tumor mutational burden (TMB) was compared between the LR and HR score groups. The clinical and transcriptome data of the IMvigor210 cohort were acquired from a freely available software and data package (http://research-pub.gene.com/IMvigor210CoreBiologies). The anti-PD-L1 treated advanced urothelial carcinoma patient’s dataset was utilized to assess the predictive capability of the PRGs-DEGs scoring system for immunotherapy response. The proportions of various immunotherapy responses, including the stable disease (SD), partial response (PR), complete response (CR), and progressive disease (PD). Moreover, the survival differences between the LR and HR subgroups were compared.

### 2.9 Establishment and validation of a nomogram for overall survival

According to the independent predictive factors such as the PRGs-DEGs scoring system (*p < 0.05*), a nomogram risk score for OS was constructed using the R “rms” package. Then, the calibration curve analysis, ROC, and decision curves analysis were carried out to elucidate the performance of the nomogram scoring system. Furthermore, calibration curves were plotted for the survival probability at 1-, 3-, and 5-year to elucidate the precision of the combined model. The clinical utility of each predictive variable was assessed via decision curve analysis. Additionally, AUC values of ROC curves were utilized to assess the reliability of each single predictive variable and the combined nomogram model.

### 2.10 Statistical analyses

For statistical measurement, the R software (version 4.2.5) was employed. Correlations among variables were analyzed by Pearson or Spearman coefficient. The intergroup differences in continuous variables were compared via the *t*-test. Based on the Kaplan-Meier method, the survival curves were drawn. Furthermore, the ROC curves were employed to assess the accuracy of PRGs-DEGs risk score for predicting survival and PRGs clusters. All the statistical measurements were two-sided, and *p < 0.05* was set as the significance level. The Wilcoxon and Kruskal–Wallis analyses were conducted to compare two or more clusters, respectively. The log-rank method was employed for Kaplan-Meier survival analysis to assess the statistical significance. Moreover, for Lasso Cox regression analysis, the R “glmnet” package was utilized. AUC values of ROC curves in different cohorts were calculated using the R “timeROC” package.

## 3 Results

### 3.1 Genetic and transcriptional landscape of PRGs in BLCA

**FIGURE 2:**
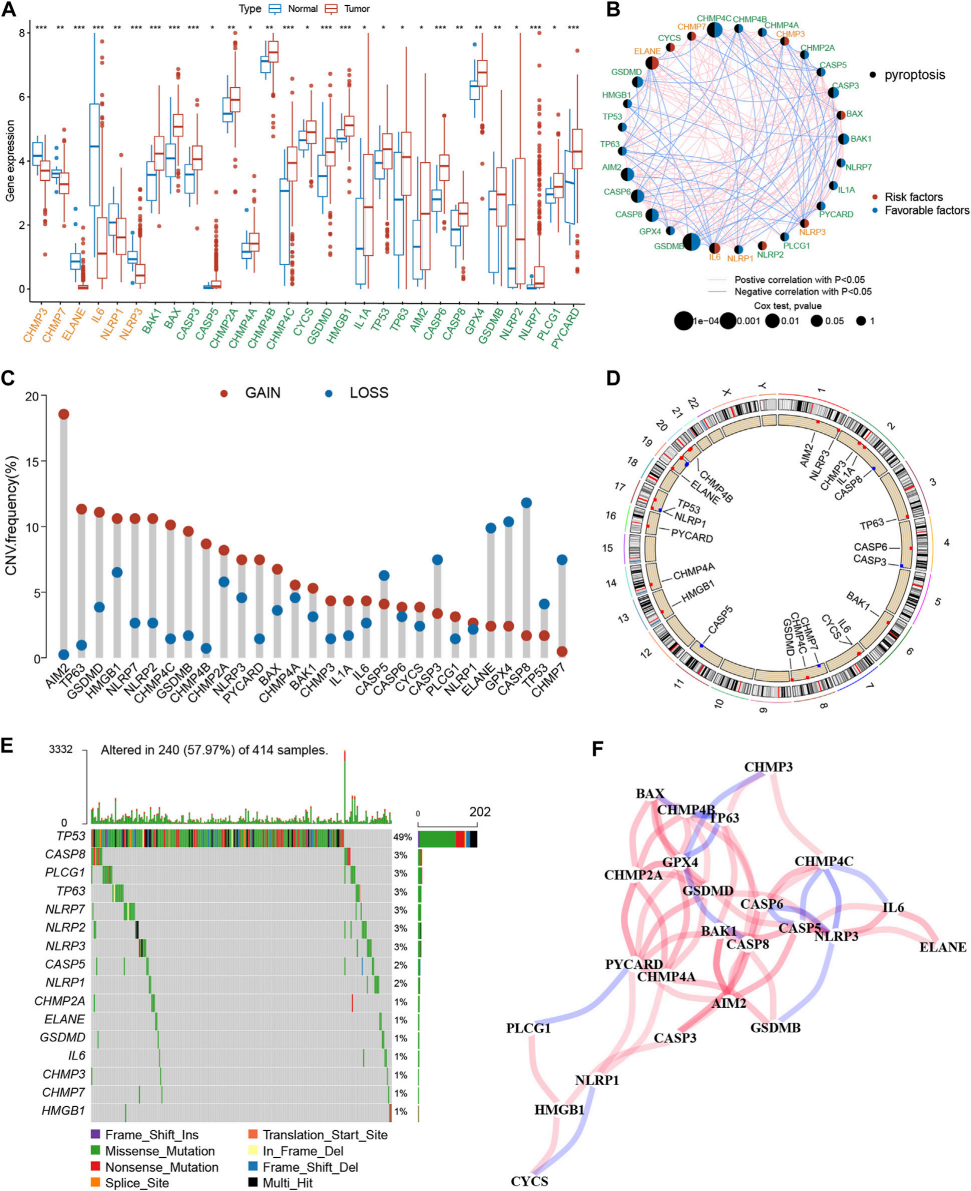
Genetic and transcriptional alterations of PRGs in BLCA. **(A)** The expression difference of 29 PRGs between normal tissue and BLCA tissue. **(B)** Interactions among PRGs in BLCA. **(C)** The CNV variation frequency of PRGs. Red circle: amplified frequency; blue circle: missing frequency. **(D)** Locations of CNV alterations in PRGs on 23 chromosomes. **(E)** Mutation frequencies of PRGs in the patients with BLCA from the TCGA cohort. **(F)** The correlation network of the PRGs. The asterisk represents the statistical *p* value (*p < 0.05; **p < 0.01; ***p < 0.001). PRGs, pyroptosis-related genes; BLCA, bladder cancer; TCGA, The Cancer Genome Atlas; CNV, copy number variant.

The expression profiles of 52 PRGs were compared in the TCGA-BLCA cohort, and 29 DEGs were assessed between the tumor and adjacent tissues (Figure 2A). Furthermore, a pyroptosis network was constructed to illustrate the comprehensive profile of PRGs interactions, modulator associations, and their prognostic value for BLCA (Figure 2B). The gain or loss copy number variation (CNV) was very common in DEGs. For example, the frequency of gain CNV in AIM2 was up to 18.5%, and that of loss CNV in CASP8 was up to 11.1% (Figure 2C). Figure 2D demonstrates CNV alterations of the PRGs on the chromosome. Moreover, the somatic mutation of these DEGs in the TCGA-BLCA cohort was also described. It was revealed that TP53 had the highest mutation frequency (49%); however, the mutation frequencies of other DEGs were all <3% (Figure 2E). The correlation network between PRGs is shown in Figure 2F. Additionally, a notable difference was observed between the expression levels and genetic profile of PRGs of BLCA and control samples, suggesting the potential role of PRGs in BLCA oncogenesis and development.

### 3.2 PRGs-based identification of molecular subtypes

**FIGURE 3:**
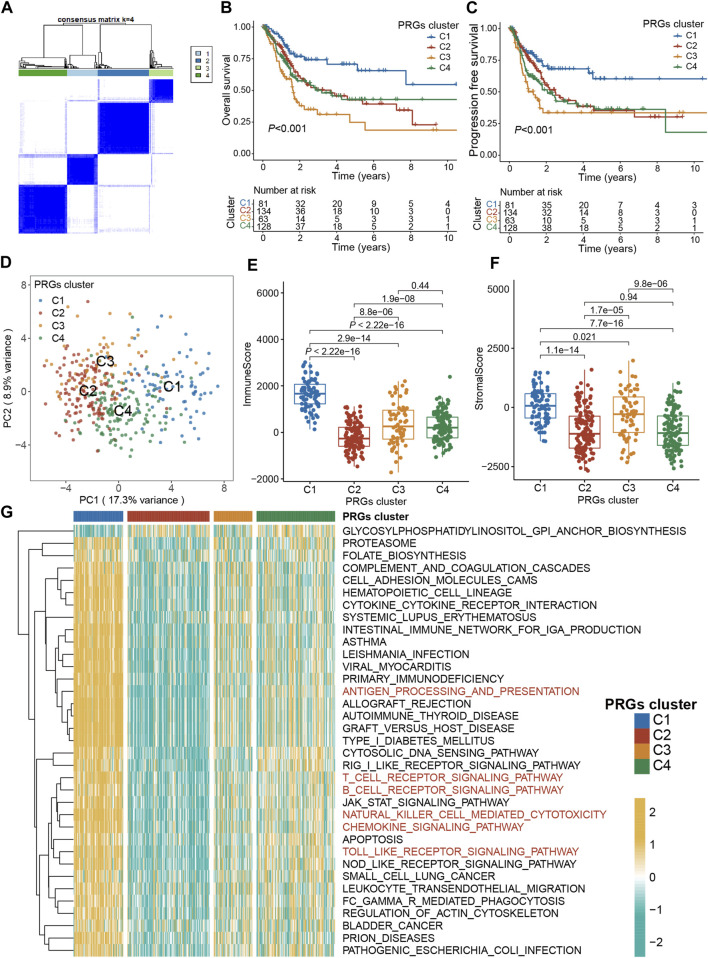
Pyroptosis subtypes and clinicopathological and biological characteristics of four distinct subtypes of samples divided by consistent clustering. **(A)** Consensus matrix heatmap defining four clusters (*k* = 4) and their correlation area. **(B)** The OS Kaplan-Meier curve of different clusters in BLCA patients. **(C)** The PFS Kaplan-Meier curve of different clusters in BLCA patients. **(D)** PCA analysis showing a remarkable difference in transcriptomes between the four subtypes. **(E)** The distribution of immune score, and **(F)** stromal score inferred by ESTIMATE algorithm between the four clusters in the TCGA BLCA cohort. **(G)** The heatmap showing the results of GSVA enrichment analysis among different pyroptosis clusters. The asterisk represents the statistical *p* value (*p < 0.05; **p < 0.01; ***p < 0.001). PRG, pyroptosis-related gene; BLCA, bladder cancer; OS, overall survival; PFS, progression-free survival; GSVA, gene set variation analysis.

To explore the association between expression profiles of DEGs and BLCA subtypes, consensus cluster analysis was performed on TCGA-BLCA patients. The increase of clustering variable (*k*) from 2 to 10, indicated that at *k* = 4, the intra-group associations were the highest, while the intergroup associations were lowest, suggesting that the TCGA-BLCA patients could be grouped into four clusters according to the expression of PRGs (Figure 3A). The expressions of PRGs in the four clusters were shown in Supplementary Figures S1A, B. Kaplan-Meier curve for PFS and OS among the four clusters indicated that patients in cluster C1 had the best prognosis, while those in C3 had the worst prognosis (*p < 0.001*, Figures 3B, C). Based on the expression profiles of DEGs, patients in four subtypes were easily distinguished (Figure 3D). Moreover, the “ESTIMATE” algorithm was employed to elucidate the stromal and immune scores of patients, which revealed that cluster C1 had the highest immune and stromal scores, proving that tumor tissues from cluster C1 patients were infiltrated by more immune cells (Figure 3E) and by more fibro-blasts/endothelial cells (Figure 3F). Additionally, the transcriptomic matrix was transformed into a pathway matrix via the GSVA algorithm, and then the correlation of PRGs clusters with KEGG pathways was assessed. Different immune-related pathways were observed to be activated in cluster C1 (Figure 3G), including the T cell receptor signaling pathway, antigen processing-presentation, B cell receptor signaling pathway, Chemokine signaling pathway, Natural killer cell-mediated cytotoxicity, and Toll-like receptor signaling pathway.

### 3.3 Infiltrating immune cells and identification of feature genes related to PRGs clusters

**FIGURE 4:**
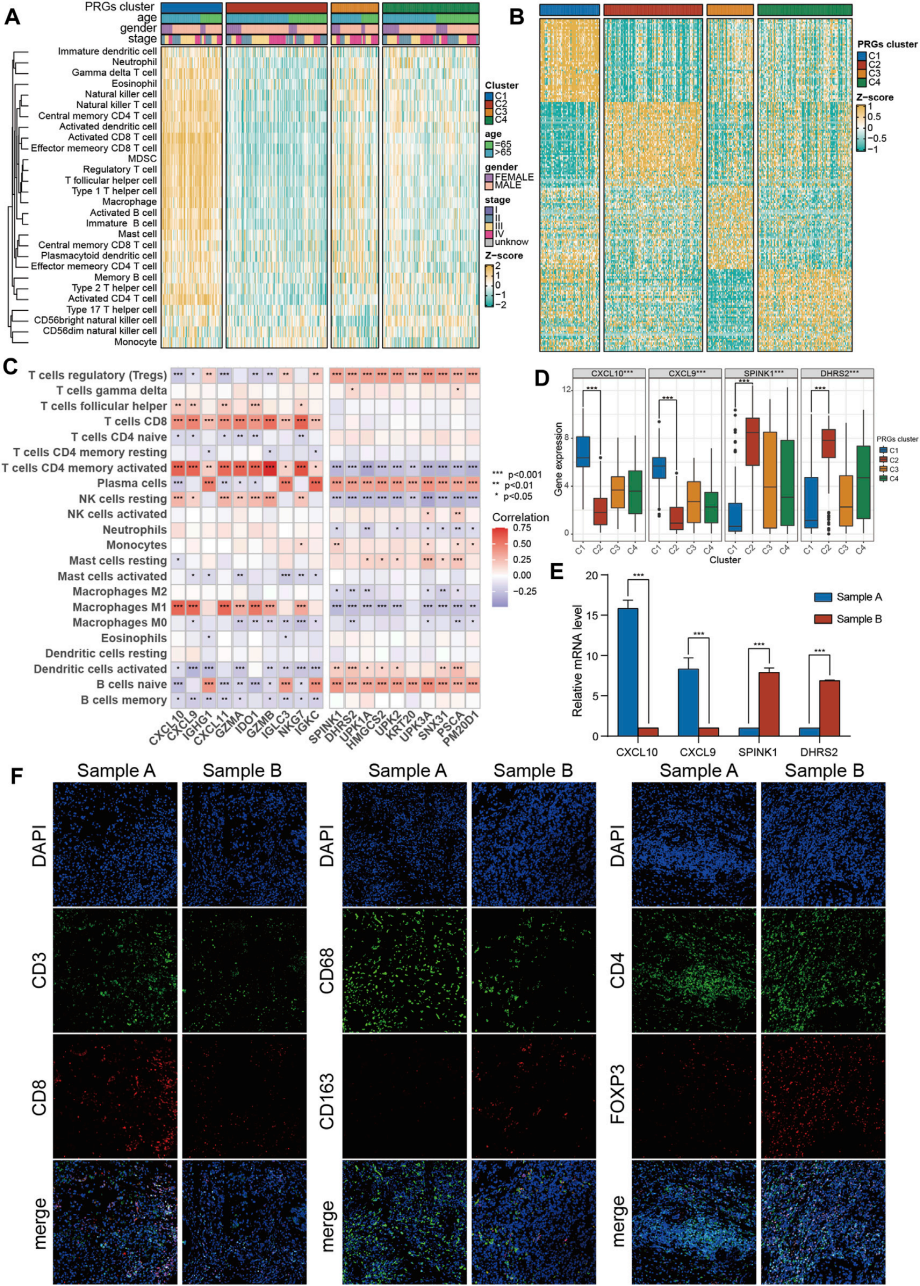
The immune features and marker genes of the four molecular clusters. **(A)** The infiltration abundance of 28 immune cell subsets evaluated by ssGSEA for four clusters. **(B)** Heatmap of differentially expressed genes for four clusters. **(C)** The association between the abundance of immune cells and the most significantly differentially expressed genes in the four clusters. **(D)** Expression of C1 and C2 cluster marker genes in the four clusters. **(E)** The expression of C1 and C2 subtype marker genes in tumor samples was detected by PCR. **(F)** The infiltration of CD8 + T cells, macrophages and Tregs in tumor samples was detected by immunofluorescence. The asterisk represents the statistical *p* value (*p < 0.05; **p < 0.01; ***p < 0.001). ssGSEA, single sample gene set enrichment analysis.

First, the transcriptomic data of all genes were transformed into scores of 28 infiltrating immune cells using the ssGSEA algorithm from the R “gsva” package, and then the differential analysis of these scores among four PRGs clusters was performed. Surprisingly, ssGSEA scores of almost all immune cells in cluster C1 were substantially increased than the cluster C2 (Figure 4A; Supplementary Figure S1C). Therefore, C1 was defined as an “immune-hot” tumor and C2 as an “immune-cold” tumor. Moreover, the differential expression assessment genes linked with immune checkpoints among four clusters showed that CD274, PDCD1, CTLA4, LAG3, and TIGIT were expressed at the highest level in cluster C1 (Supplementary Figure S1D).

To promote the clinical application of novel prognostic biomarkers, key PRGs characteristic of each cluster were identified. First, using a heatmap of differentially expressed genes was employed to characterize four subtypes (Figure 4B). Then, the correlation of feature genes of C1 and C2 with ssGSEA scores of 28 immune cells was assessed. It was revealed that feature genes of the C1 subtype (immune-hot) were predominantly positively correlated with activated CD4 T cells, M1 macrophages, and CD8 T cells, while those of the C2 subtype (immune-cold) were mainly positively correlated with Tregs (Figure 4C). Among features genes, CXCL9/CXCL10 were upregulated in C1 and downregulated in C2, while SPINK1/DHES9 were upregulated in C2 and downregulated in C1 (Figure 4D). Furthermore, the expression levels of these features genes of C1 (CXCL9/CXCL10) and C2 (SPINK1/DHES9) in tumor tissues were validated and one C1 (sample A, CXCL9/CXCL10 high + SPINK1/DHES9 low) and one C2 (sample B, SPINK1/DHES9 high + CXCL9/CXCL10 low) sample were screened for subsequent immunofluorescence assay (Figure 4E). The detailed clinical features of the two BLCA patients are shown in Supplementary Table S4. Consistent with bioinformatics analysis, sample A had abundant infiltration of CD8 + T cells and CD68 + macrophages, which corresponded to C1 features, while sample B with high SPINK1 and DHRS2 expression had notably more M2 macrophages and Treg infiltration, which corresponded to C2 subtype features (Figure 4F).

### 3.4 Differentially expressed genes-based identification of molecular subtypes

**FIGURE 5:**
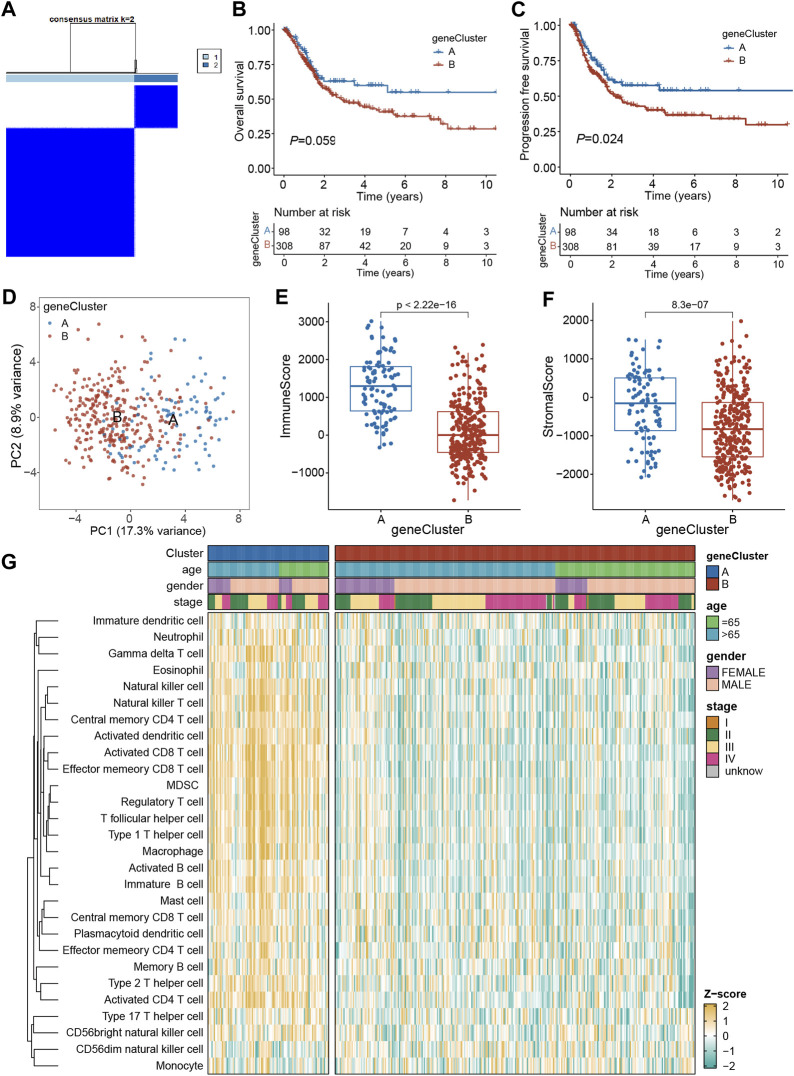
Identification of geneClusters based on PRGs-DEGs. **(A)** Consensus matrix heatmap defining two clusters (*k* = 2) and their correlation area. **(B)** Kaplan-Meier curves for OS of the two geneClusters. **(C)** Kaplan-Meier curves for PFS of the two geneClusters. **(D)** PCA analysis showing a remarkable difference in transcriptomes between the two geneClusters. **(E)** The distribution of immune score, and **(F)** stromal score inferred by ESTIMATE algorithm between the two geneClusters in the TCGA BLCA cohort. **(G)** The infiltration abundance of 28 immune cell subsets evaluated by ssGSEA for the two geneClusters. The asterisk represents the statistical *p* value (*p < 0.05; **p < 0.01; ***p < 0.001). DEGs, differentially expressed genes; OS, overall survival; PFS, progression-free survival; PRGs, pyroptosis-related genes.

To assess the underlying genetic alterations, first, 240 DEGs (PRGs-DEGs) among four clusters were identified. Then, based on these genes, unsupervised clustering was carried out to categorize TCGA-BLCA patients into geneClusters A (*n* = 98) and B (*n* = 308) (Figure 5A). Kaplan-Meier curve revealed that geneCluster A patients had longer OS and PFS than geneCluster B patients, although the difference in OS was not significant (*p = 0.059*) (Figures 5B, C). Moreover, PCA analysis also validated that both geneClusters were well distinguishable by PRGs-DEGs (Figure 5D). Additionally, the immune and stromal scores between the two geneClusters were assessed, which indicated that immune and stromal scores in geneCluster A were both markedly increased than geneCluster B (*p < 0.001*) (Figures 5E, F). This might be why geneCluster A patients had longer OS and PFS than geneCluster B patients. Furthermore, ssGSEA scores of almost all infiltrating immune cells in geneCluster A were remarkably higher than those in geneCluster B (Figure 5G).

### 3.5 Construction and validation of PRGs-DEGs risk scoring system

**FIGURE 6:**
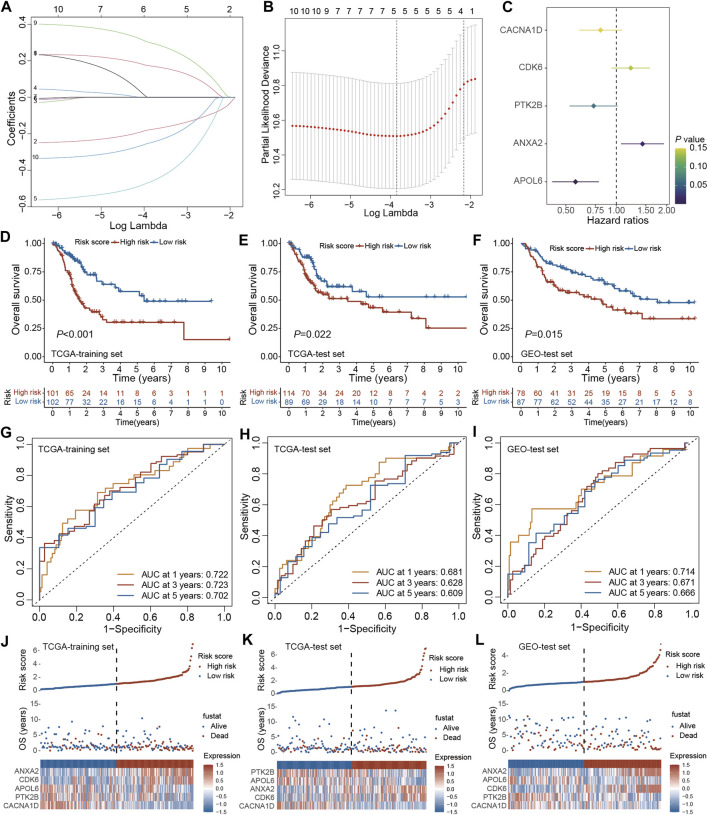
Construction and validation of the PRGs-DEGs risk score in the training and test set. **(A,B)** The LASSO method of PRGs associated with prognosis. **(C)** Forrest plot of the multivariate Cox regression analysis of five genes. **(D)** Kaplan–Meier curves of survival in TCGA training set. **(E)** Kaplan–Meier curves of survival in TCGA test set. **(F)** Kaplan–Meier curves of survival in GEO test set. **(G)** Time-dependent ROC curve of the risk score model for predicting 1, 3, 5 years in TCGA training set. **(H)** Time-dependent ROC curve of the risk score model for predicting 1, 3, 5 years in TCGA test set. **(I)** Time-dependent ROC curve of the risk score model for predicting 1, 3, 5 years in GEO test set. **(J)** The distribution, survival status, and heat map of risk scores in the TCGA training set. **(K)** The distribution, survival status, and heat map of risk scores in the TCGA test set. **(L)** The distribution, survival status, and heat map of risk scores in the GEO test set. The asterisk represents the statistical *p* value (*p < 0.05; **p < 0.01; ***p < 0.001). PRGs, pyroptosis-related genes; LASSO, least absolute shrinkage and selection operator; PCA, principal component analysis; OS, overall survival; ROC, receiver operating characteristic.

The prognostic model was established using the TCGA training set and its performance was evaluated through internal testing with the TCGA test set and external testing with the GEO test set. Based on 240 PRGs-DEGs, the risk scoring system was generated using the TCGA-BLCA train cohort. The univariate Cox regression analysis identified 46 survival-related genes, which were further screened to 10 by LASSO regression analysis (Figures 6A, B). Subsequently, the predictive model was generated using the multivariate Cox regression analysis, and 5 genes were identified. According to the hazard ratio in the model, CACNA1D, PTK2B, and APOL6 were tumor suppressor genes, while CDK6 and ANXA2 were oncogenes (Figure 6C). The PRGs score was calculated as follows: PRGs score = (−0.216021228 × CACNA1D) + (0.199209604 × CDK6) + (−0.310925889 × PTK2B) + (0.360364278 × ANXA2) + (−0.56043276 × APOL6).

The patients were then categorized as LR and HR cohorts based on the median PRGs score. PCA analysis confirmed that the PRGs score based on the above five genes could well distinguish the two risk groups (Supplementary Figure S2A). Moreover, the correlation analysis also validated that PRGs score was negatively linked with tumor suppressor genes (CACNA1D, PTK2B, and APOL6) and positively linked with oncogenes (CDK6 and ANXA2) (Supplementary Figures S2B–F). The expression of PRGs between the HR and LR groups is illustrated in Supplementary Figure S2G. It was observed that BLCA patients’ prognosis in the LR cohort was better than the HR cohort in both the training and internal test sets (Figures 6D, E). ROC analysis revealed that AUC for 1/3/5 years OS was 0.722/0.723/0.702 for the training set and 0.681/0.628/0.609 for the internal test set, respectively (Figures 6G, H). Additionally, with the help of the heatmap, the expression levels of the 5 PRGs of the prognostic model in the LR and HR cohorts were visualized (Figures 6J, K). It was validated that the constructed model could help predict the outcomes of BLCA patients.

For validating the prognostic model in the external test set, each patient’s PRGs score was assessed based on the aforementioned PRGs score formula. The external test set patients were categorized into the LR and HR cohorts based on the training set’s median PRGs score value. In line with the data acquired for the training set, the HR group patients in the external test set indicated a poorer prognosis than the LR group patients (Figure 6F). Additionally, the ROC analysis revealed an AUC of 0.714/0.671/0.666 for 1/3/5 years OS (Figure 6I). Figure 6L demonstrates the survival status and the heatmap of these 5 prognostic genes in the external test set. Overall, these results indicated that the constructed prognostic model could accurately predict a BLCA patient’s prognosis from the external test set.

**FIGURE 7:**
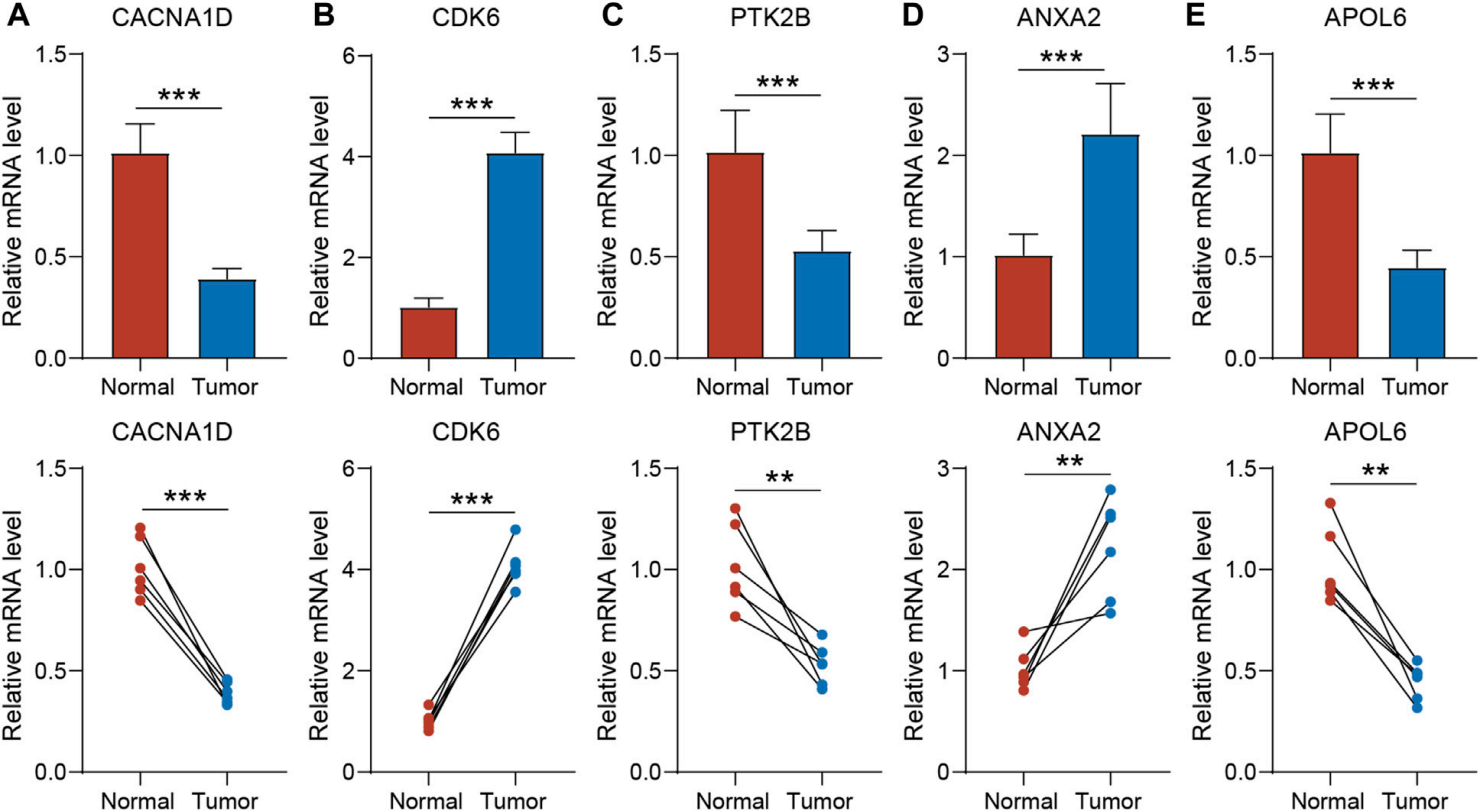
The expression of CACNA1D **(A)**, CDK6 **(B)**, PTK2B **(C)** ANXA2 **(D)** and APOL6 **(E)** in normal bladder tissue and BLCA tissue of patients. *t*-test was used to compare the expression of genes between normal and tumor. The asterisk represents the statistical *p* value (*p < 0.05; **p < 0.01; ***p < 0.001). BLCA, bladder cancer.

To validate the expression of the five genes involved in the risk signature in BLCA patients, we collected clinical BLCA samples and paired normal tissues, and analyzed them using qPCR. As demonstrated in Figure 7, CDK6 and ANXA2 exhibited elevated expression levels in tumors, whereas CACNA1D, PTK2B, and APOL6 exhibited significantly reduced expression levels in tumors. These distinctions align with our bioinformatic findings, suggesting that these genes may serve as innovative biomarkers for prognostic prediction of BLCA.

### 3.6 The association of PRGs-DEGs risk score with clinicopathologic characteristics

**FIGURE 8:**
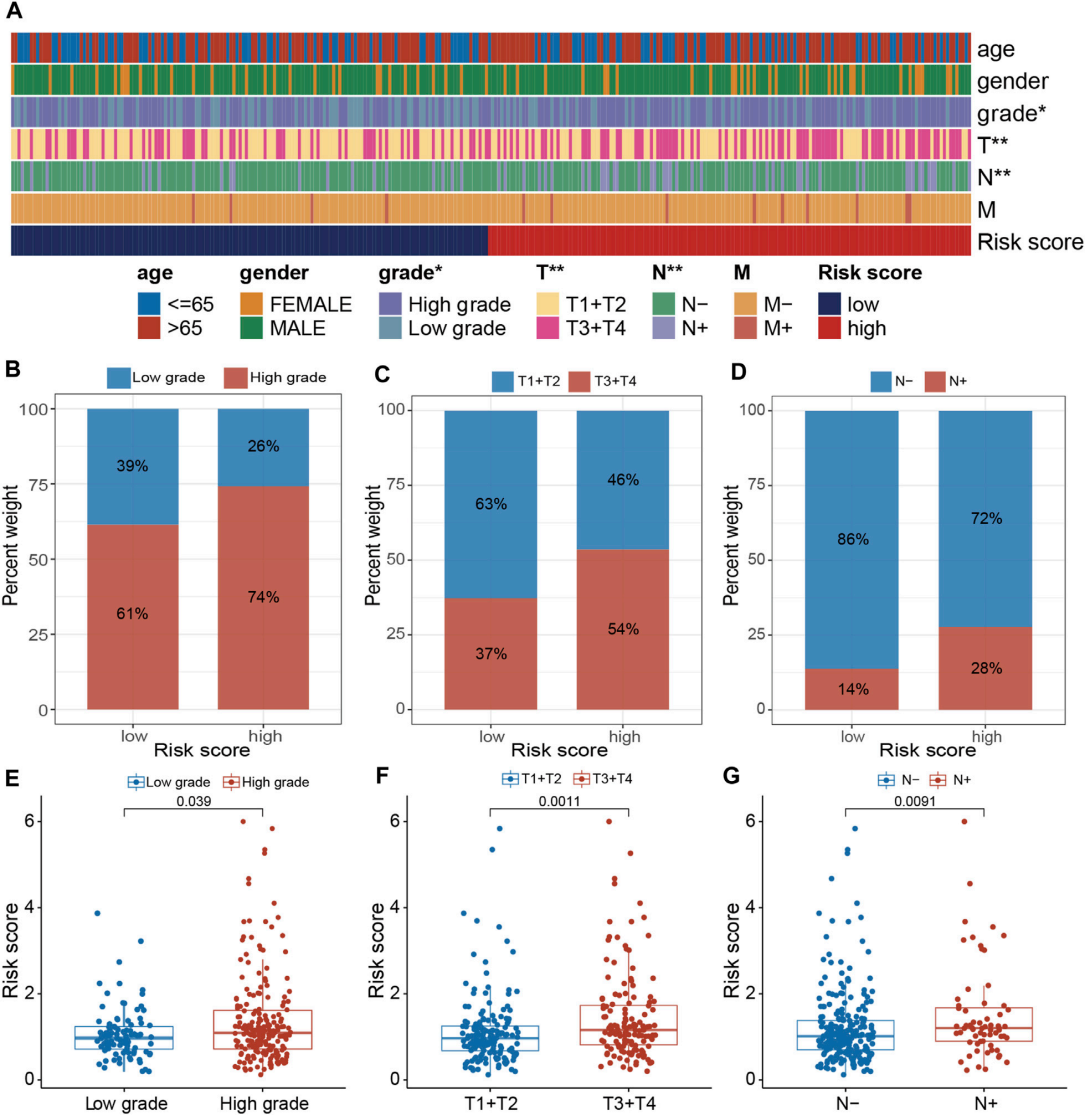
Clinical evaluation of the panel by PRGs-DEGs risk score. **(A)** A band chart of risk score and clinical features of BLCA patients. **(B)** The proportion of patients with different grade category in high- and low-risk groups. **(C)** Comparison of the risk score between the patients with different grade category (*p* = 0.039, Wilcoxon test). **(D)** The proportion of patients with different T category in high- and low-risk groups. **(E)** Comparison of the risk score between the patients with different T category (*p* = 0.0011, Wilcoxon test). **(F)** The proportion of patients with different N category in high- and low-risk groups. **(G)** Comparison of the risk score between the patients with different N category (*p* = 0.0091, Wilcoxon test). The asterisk represents the statistical *p* value (*p < 0.05; **p < 0.01; ***p < 0.001). BLCA, bladder cancer.

The clinical relevance of the PRGs-DEGs risk model was assessed. The chi-square test was carried out to elucidate the differences in clinicopathological features between LR and HR subgroups. The heatmap indicates that the pathologic T stage, tumor grade, and pathologic N stage were closely linked with the PRGs score (*p < 0.001*) (Figure 8A). Furthermore, the proportions of high tumor grade, pathologic T3 + T4 stage and lymph node (+) in the HR subgroup were substantially greater than in the LR subgroup, whereas proportions of low tumor grade, pathologic T1 + T2 stage, and lymph node (−) in the HR subgroup were markedly reduced than in LR subgroup (Figures 8B–D). Additionally, the difference in PRGs score among distinct sub-groups was assessed based on clinicopathological characteristics. It was revealed that the PRGs score in high-grade, T3 + T4, and lymph node (+) subgroups were remarkably higher than those in low-grade, T1 + T2, and lymph node (−) subgroups (Figures 8E–G). To explore whether the PRGs score applies to different clinical subgroups, Kaplan-Meier curves curves were used to assess the presence of prognosis differences between LR and HR groups among diverse clinical groups. Markedly significant differences were observed between the HR and LR cohorts in the age ≤ 65, age > 65, female, male, low grade, high grade, T1-2, T3-4, N0, N1-3, and M0 groups. Overall, compared with HR, the LR cohort had a significant survival advantage (Supplementary Figure S3).

### 3.7 Correlation analysis of PRGs-DEGs risk score with oncogenic pathways and immune cells

**FIGURE 9:**
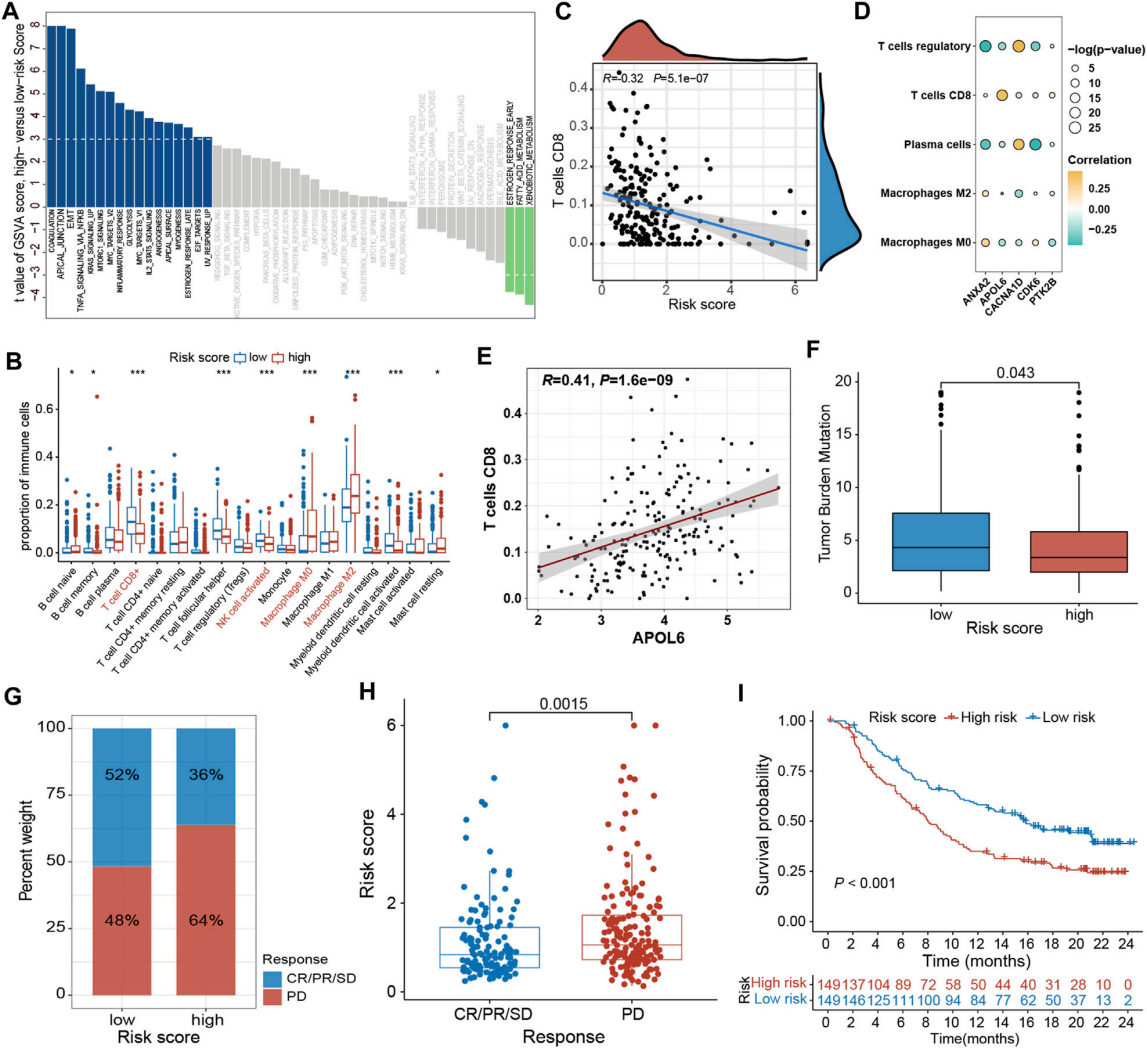
Comprehensive analysis of the PRGs-DEGs risk score in BLCA. **(A)** Differences in biological function between high- and low-risk groups. **(B)** The differences of immune cells between high- and low-risk groups based on ssGSEA. **(C)** Correlations between risk score and CD8 + T cells. **(D)** Correlations between the abundance of immune cells and 5 genes in the proposed model. **(E)** Correlations between APOL6 gene and CD8 + T cells. **(F)** Differences in TMB between high- and low-risk groups. **(G)** The proportion of patients with (CR/PR/SD) or without (PD) response to PD-L1 blockade therapy in the high- and low-risk groups in the IMvigor210 cohort. **(H)** Different risk score in CR/PR/SD group and PD group in IMvigor210 cohort (*p* < 0.05). **(I)** OS curves for the high- and low-risk groups in IMvigor210 cohort (*p* = 0.002). The asterisk represents the statistical *p* value (*p < 0.05; **p < 0.01; ***p < 0.001). BLCA, bladder cancer; ssGSEA, single sample gene set enrichment analysis; TMB, tumor mutation burden; OS, overall survival; SD, stable disease; PD, progressive disease; CR, complete response; PR, partial response.

To elucidate the mechanism by which the risk signature affects BLCA initiation and progression, the relation of PRGs score with the hallmark oncogenic pathways and infiltrating immune cells was assessed. The differential analysis of the ssGSEA score of hallmark pathways revealed 20 of 50 items, which were remarkably altered between LR and HR subgroups. Specifically, epithelial-mesenchymal transition, KRAS signaling, mtorc1 signaling, and TNFα signaling via NF-κB were greatly enriched in the HR subgroup (Figure 9A; Supplementary Figure S4A). Additionally, the proportions of intratumoral immune cells were quantified via the CIBERSORT algorithm. The proportions of NK and CD8 + T cells were substantially increased in the LR subgroup than in the HR subgroup, while opposite data was acquired for M2-type macrophages (type of suppressive immune cells) (Figure 9B; Supplementary Figure S4B). In particular, the PRGs score was markedly negatively linked with CD8 + T cells (Figure 9C; Supplementary Figure S5). Moreover, the relationship between these 5 signature genes and immune cell abundance was also elucidated, which indicated Tregs, plasma cells, CD8 + T cells, as well as M2- and M0-type macrophages were notably correlated with these genes (Figure 9D). Particularly, the APOL6 gene was markedly positively linked with CD8 T cell infiltration (Figure 9E).

### 3.8 The role of the PRGs-DEGs risk score in predicting immunotherapy response

Accumulating evidence has shown that high TMB patients benefit from immunotherapy because of enhanced neoantigens. Our genomic data analysis of the TCGA-BLCA cohort indicated a lower TMB in the HR subgroup than in the LR subgroup (*p = 0.043*; Figure 9F), implying that LR subgroup patients are more likely to benefit from anti-PD-1/PD-L1 immunotherapy. Moreover, a public dataset IMvigor210 cohort was also analyzed to ensure the predictive significance of PRGs score in immunotherapy. Individuals were classified into LR and HR subgroups based on the median score. It was noticed that the proportion of responders (CR/PR/SD) in the LR subgroup was notably increased than in the HR subgroup, whereas the proportion of non-responders (PD) was substantially reduced in the LR subgroup than in the HR subgroup (Figure 9G; Supplementary Figures S4C, E). The PRGs score in the HR subgroups was markedly higher than that in the LR subgroup (*p = 0.0015*, Figure 9H; Supplementary Figures S4D, F). In addition, the Kaplan-Meier survival curve indicated that HR patients had a shorter OS than the LR patients (*p < 0.001*, Figure 9I).

### 3.9 The nomogram based on clinical characteristics and PRGs-DEGs risk score

**FIGURE 10:**
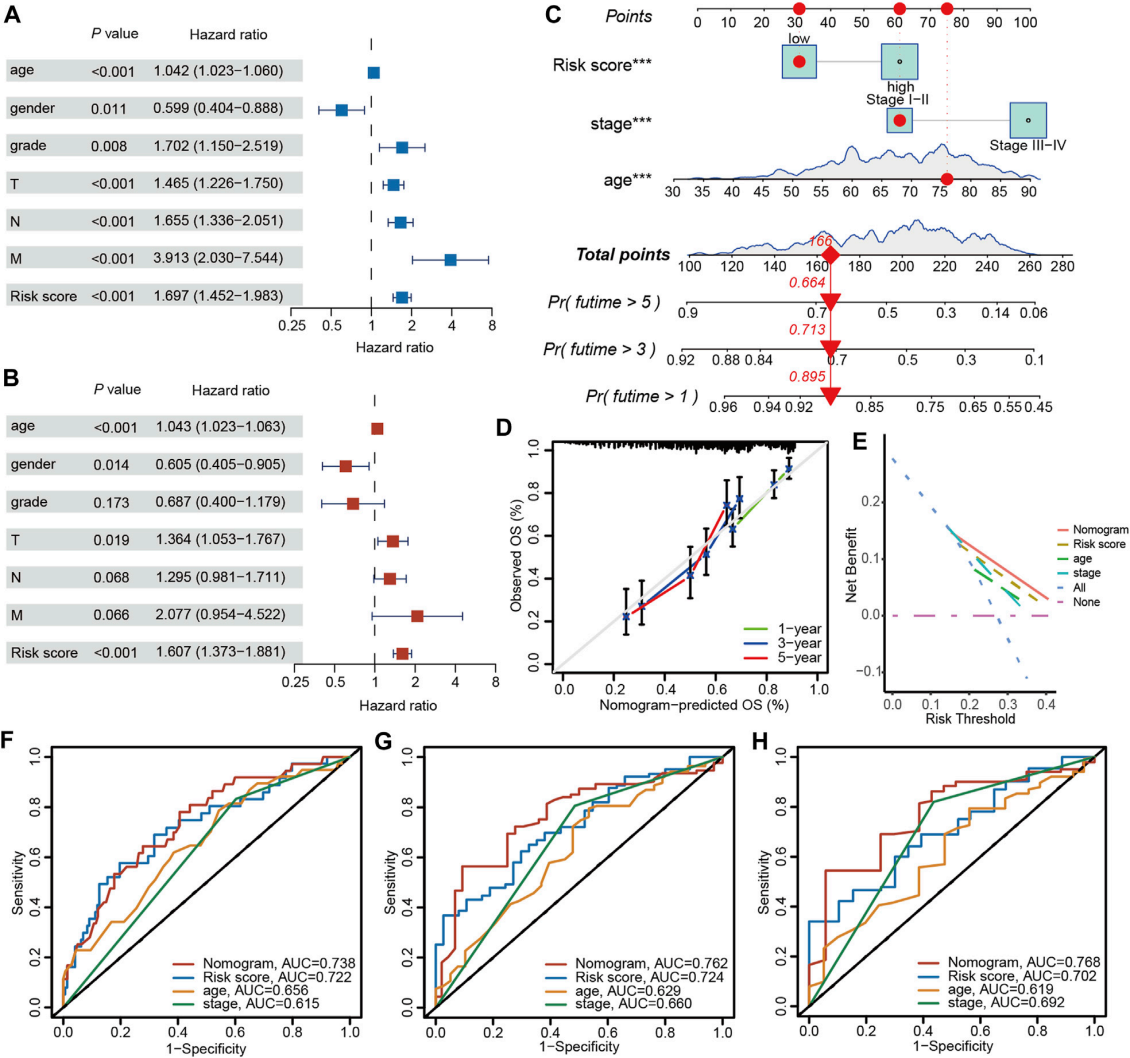
Nomogram construction and prognostic value of the signature. **(A)** Univariate and **(B)** Multivariate Cox regression analysis of clinical factors and risk score. **(C)** The nomogram for predicting the survival rate of 1-, 3-, and 5-years in BLCA patients. **(D)** Calibration plots of the nomogram. **(E)** Decision curve analysis of the nomogram of the panel. The time-dependent ROC analysis of nomogram predicting the survival rate of 1-years **(F)**, 3-years **(G)**, and 5-years **(H)** in BLCA patients. The asterisk represents the statistical *p* value (*p < 0.05; **p < 0.01; ***p < 0.001). BLCA, bladder cancer; ROC, receiver operating characteristic.

Univariate and multivariable Cox regression analyses were carried out to elucidate independent prognostic factors in BLCA patients. The univariate Cox regression analysis revealed that PRGs score and most clinical parameters were prognostic factors (Figure 10A); however, multivariate Cox regression indicated that only PRGs score, pathologic T stage, and age were independent prognostic factors for OS (Figure 10B). Therefore, according to the PRGs score, tumor stage, and age, a nomogram was generated to predict BLCA patients’ 1-, 3-, and 5-year survival probability (Figure 10C). One point was given to each patient for each prognostic parameter, and higher total points depicted a worse outcome. Moreover, calibration plots revealed that the nomogram had a similar performance to an ideal model (Figure 10D). Additionally, ROC and DCA data also illustrated that the nomogram had a high efficiency for clinical implementation (Figures 10E–H).

## 4 Discussion

Much literature has indicated the essential activity of pyroptosis in antitumor mechanisms and innate immunity (Wang et al., 2020; Tsuchiya, 2021). However, most of these researches were focused on a single TME cell or PRG; therefore, the overall influence and TME infiltration characteristics regulated by the simultaneous influence of different PRGs remain undetermined. This investigation indicated global transcriptional and genetic level changes of PRGs in BLCA. Here, four distinct PRGs clusters were identified based on 52 PRGs. Subtype C1 patients had the highest OS and PFS than other subtypes. Furthermore, by analyzing the differences in the TME between four clusters, we found that PRGs subtype C1 and C2 showed distinct and typical characteristics. Specifically, PRGs subtype C1 showed an “immune-hot” phenotype, which was characterized by substantial immune activation, such as antigen presentation and processing, natural killer cell-induced cytotoxicity, the B and T-cell receptor signaling pathways, the JAK-STAT signaling pathway, and the NOD-like, Toll-like, and RIG-I-like receptor signaling pathways; however, PRGs subtype C2 showed “immune-cold” characteristics. Additionally, two geneClusters were also identified according to the DEGs between the PRGs clusters. Therefore, the results of this study revealed that PRGs are a potential predictor for elucidating BLCA’s clinical outcomes and immunotherapy response. Thus, a robust and efficient prognostic PRGs-DEGs risk score model was established and its predictive ability was assessed. The pyroptosis patterns characterized by immune suppression and stimulation indicated HR and LR scores, respectively. The LR and HR patients indicated markedly different clinicopathological features, mutation, prognosis, immune checkpoints, TME, and anti-PD1/PD-L1 immunotherapy susceptibilities. Lastly, by integrating risk score, stage, and age, a quantitative nomogram was established, further improving the model’s performance and facilitating the application of the risk score. After construction and validation, our prediction model, compared to previous models, can not only predict the prognosis of BLCA but also assess the tumor immune microenvironment and the efficacy of immunotherapy. This provides valuable diagnostic and therapeutic assistance to clinicians. The prognostic model can be employed for prognostic stratification of BLCA patients, assists in better identification of BLCA molecular pathways, and provides novel strategies for targeted therapies.

Pyroptosis is observed in pathogen-infected cells as a programmed mechanism of death and thus stimulates the body’s inflammatory response (Bedoui et al., 2020). Under pathogenic stimulation, apoptosis can transform into pyroptosis. Furthermore, pyroptosis has been associated with different pathways in various cancers. Moreover, it has been indicated to inhibit tumor growth in liver, colorectal, and skin cancers (Zaki et al., 2010; Ellis et al., 2011; Ma et al., 2016); however, it has a two-way impact on breast cancer (Chen et al., 2012). Therefore, assessing the prognostic value based on the levels of different gasdermins alone is controversial. In BLCA patients, the association between PRGs and that between PRGs and TME remains unclear. This research investigated all the direct pathways linked with pyroptosis and elucidated a prognostic signature by assessing the impact of these pathways on TME. Currently, pyroptosis has been utilized in anti-tumor therapy, and this research suggests that it is closely linked with immunotherapy efficacy and could be employed as a biomarker for efficacy prediction.

The inhibition of immunoinhibitory molecules such as PD-1 and PD-L1 can lead to tumor regression by restoring the cytotoxicity of immune cells (Bellmunt et al., 2017). To date, several immune checkpoint inhibitors (ICIs), such as atezolizumab (PD-L1 inhibitor) and nivolumab (PD-1 inhibitor), have been approved by the FDA for the treatment of advanced BLCA (Aggen and Drake, 2017; Lobo et al., 2017). However, patient responses to ICI therapy vary greatly, with some patients achieving complete remission while others experience continuous disease progression (Jiang et al., 2020). Here, we demonstrated that PRGs can enhance anti-tumor immune responses by regulating inflammatory responses and the immune microenvironment, thereby affecting immune cell infiltration and activation in tumors. Additionally, the PRGs-DEGs risk score was significantly associated with the response of BLCA to ICI therapy, with a low-risk score indicating increased sensitivity to ICIs. This suggests that the application of the PRGs-DEGs risk score could assist in decision-making for the treatment of BLCA.

After conventional chemotherapy, BLCA prognosis is substandard, with increased levels of tumor-infiltrating lymphocytes, tumor neoantigens, and checkpoints. Although immunotherapy has undergone many advances, BLCA patients’ prognosis remains heterogeneous, suggesting that TME may play an important role. The TME comprises tumor-infiltrating immune cells (TIICs), fibroblasts, bone marrow-derived inflammatory cells, lymphocytes, blood vessels, and extracellular matrix (ECM) (Turley et al., 2015). It has been indicated that TME is essentially involved in tumor development, progression, and drug resistance (Hinshaw and Shevde, 2019). Here, the pyroptosis pattern manifested by immune inhibition (subtype C2) was linked with an HR score, while those characterized by immune activation (subtype C1) were related to an LR score. Furthermore, it was discovered that the TME characteristics and the relative abundance of 22 TIICs were substantially different between different PRGs clusters and PRGs score. These results indicated the essential role of PRGs in BLCA’s TME. Much research has indicated that effector memory T cells, T cells, and T-cell differentiation are crucially linked with immune defense in BLCA (Yang et al., 2022). The γδ-T cells can efficiently identify and kill BLCA cells, thereby inhibiting tumor progression (Nguyen et al., 2022). In addition, the density of T cells infiltrating BLCA tissue was positively correlated with prognosis (Poch et al., 2018; Bunch et al., 2020). Subtype C1 and the LR group had a better prognosis and indicated increased infiltration of activated memory CD4 + and CD8 + T cells, as well as γδ-T cells, indicating their positive involvement in BLCA prognosis. Tregs infiltration inhibits the anti-cancer immune response and has been linked with substandard prognosis (Tanaka and Sakaguchi, 2017). This is consistent with the results of the current study, where more Tregs were observed in the TME of C2 patients and the HR group.

Recent literature has indicated that B cells are also associated with the immune response (Cabrita et al., 2020; Helmink et al., 2020). Petitprez et al. (2020) suggested that B-cell enrichment was a significant prognostic factor for long-term survival and was positively linked with PD-1 blockade response in soft-tissue sarcomas. Furthermore, Helmink et al. (2020) suggested that the expression of B-cell-associated genes JCHAIN, MZB1, and IGLL5 was notably increased in patients who responded to immune checkpoint inhibitors than in non-responders. Moreover, tumor-infiltrating B cells were linked with a favorable prognosis in BLCA (Jiang et al., 2019; Zhou et al., 2021). Overall, these data suggest that B cells are not just bystanders in anti-tumor immunotherapy, instead, they offer new directions for immunotherapy and are powerful weapons against tumors. Here, a marked difference was observed in B-cell infiltration between the risk score groups and PRGs subtypes. Furthermore, naive B cell abundance in the C2 and HR groups with worse OS was notably lower than that in the C1 and the LR cohort. Therefore, B cell infiltration suppressed BLCA progression, consistent with previous literature (Jiang et al., 2019; Zhou et al., 2021).

With the development of molecular biology and tumor immunology, immunotherapy has opened new directions for treating tumors. Such therapies mainly include ICIs, cell therapy, and therapeutic antibodies. Currently, much research on ICIs for PD-1, CTLA-4, and PD-L1 is underway, and clinical trials have revealed their efficacy and safety in BLCA (Carosella et al., 2015; Farina et al., 2017; Hussain et al., 2018). This investigation identified increased levels of PD-1 and PD-L1 in the LR cohort, which showed a better response to anti-PD1/PD-L1 immunotherapy. In addition, TMB is a new essential characteristic of cancer and is related to microsatellite instability (Hatakeyama et al., 2018; Steuer and Ramalingam, 2018). In the human cancer genome, enhanced TMB is caused by a combination of endogenous factors and environmental damage (Roberts and Gordenin, 2014). It has been indicated that high TMB patients benefit better from immunotherapy (Carbone et al., 2017). Therefore, TMB has become another emerging biomarker for the prediction of the response to immunotherapy (Klebanov et al., 2019). Here, higher TMB was identified in the LR group, and the correlation analysis suggested that TMB was negatively correlated with the risk score. In addition, tremelimumab (anti-CTLA-4) has indicated good tolerance in BLCA patients who have not responded well to other immunotherapies (Chung et al., 2010). Overall, it was concluded that patients with LR scores; higher PD-1, CTLA-4, and PD-L1, expression; and increased TMB might respond well to ICIs.

## 5 Limitations

This research has certain limitations. 1) This investigation utilized data from a public database and was validated with a small clinical sample, therefore, additional *in vivo* and *in vitro* analyses and large-scale prospective research are required to validate the acquired data. 2) Some essential clinical information, including the data on neoadjuvant chemotherapy, surgery, and chemoradiotherapy, was not assessed in this study, which may affect the outcome of pyroptosis state and immune response.

## 6 Conclusion

In summary, this comprehensive investigation indicated the regulatory mechanism of PRGs, which affects the clinicopathological features, tumor’s immune-stromal microenvironment, and prognosis of BLCA patients. Furthermore, the therapeutic liability of PRGs in immunotherapy was also indicated. This research highlights the essential evidence for the clinical implications of PRGs and furnishes a novel strategy for guiding personalized immunotherapy for BLCA patients.

## Data Availability

The original contributions presented in the study are included in the article/Supplementary Material, further inquiries can be directed to the corresponding authors.
