# Supplementary material for: Prediction of immunotherapy response of bladder cancer with a pyroptosis-related signature indicating tumor immune microenvironment
Source: Front Pharmacol. 2024 Jun 25;15:1387647. doi: 10.3389/fphar.2024.1387647 (PMC11231188; doi:10.3389/fphar.2024.1387647)
Supplement: Supplementary file 1 [file DataSheet1.docx]

Supplementary Material

# Supplementary Figures and Tables

## Supplementary Figures


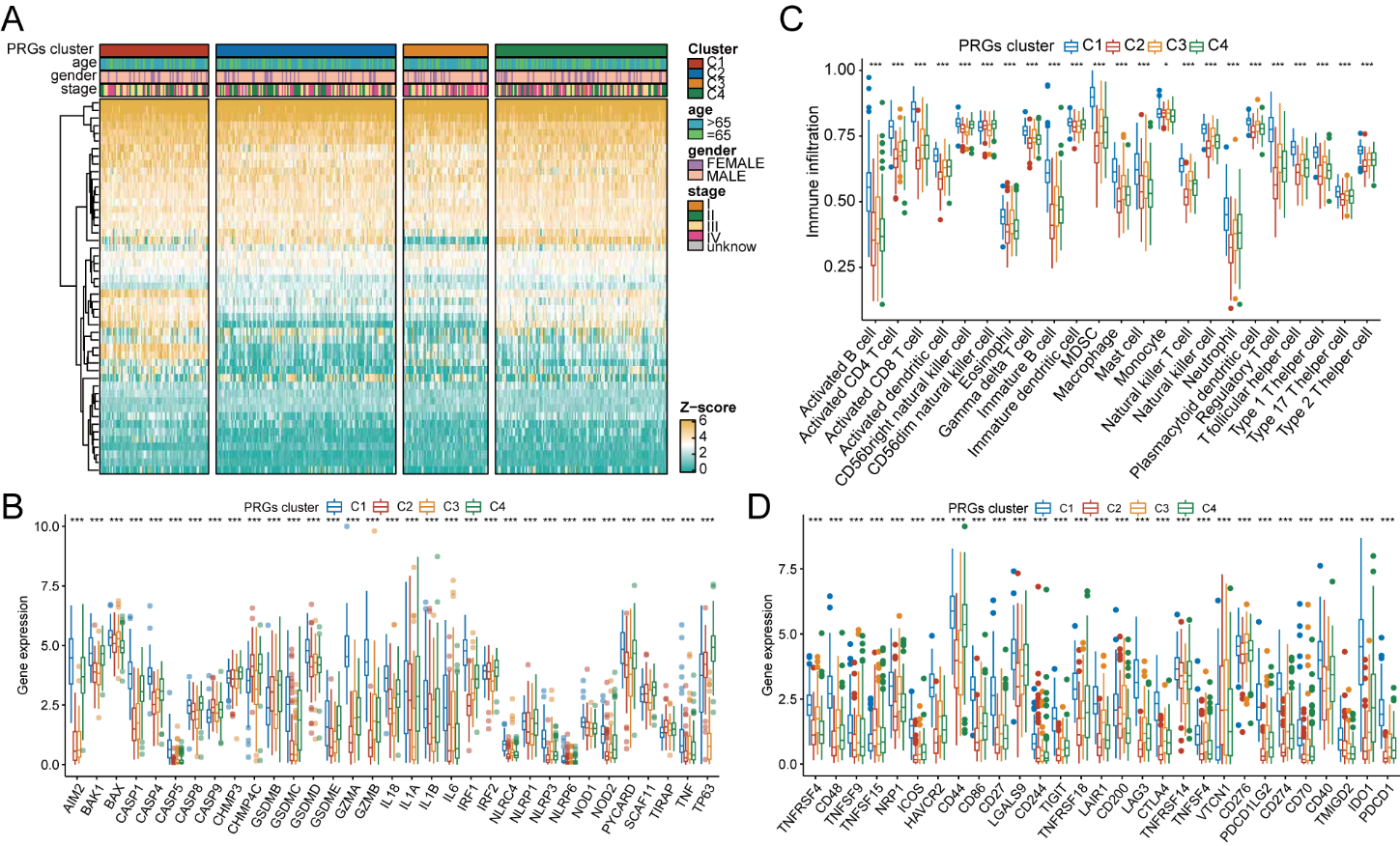


**Figure S1.** The immune features of the four molecular clusters. (A, B) Differences in the expression of PRGs among the four clusters. (C) Differences in immune cell infiltration among the four clusters. (D) Differences in the expression of immune checkpoints among the four clusters.


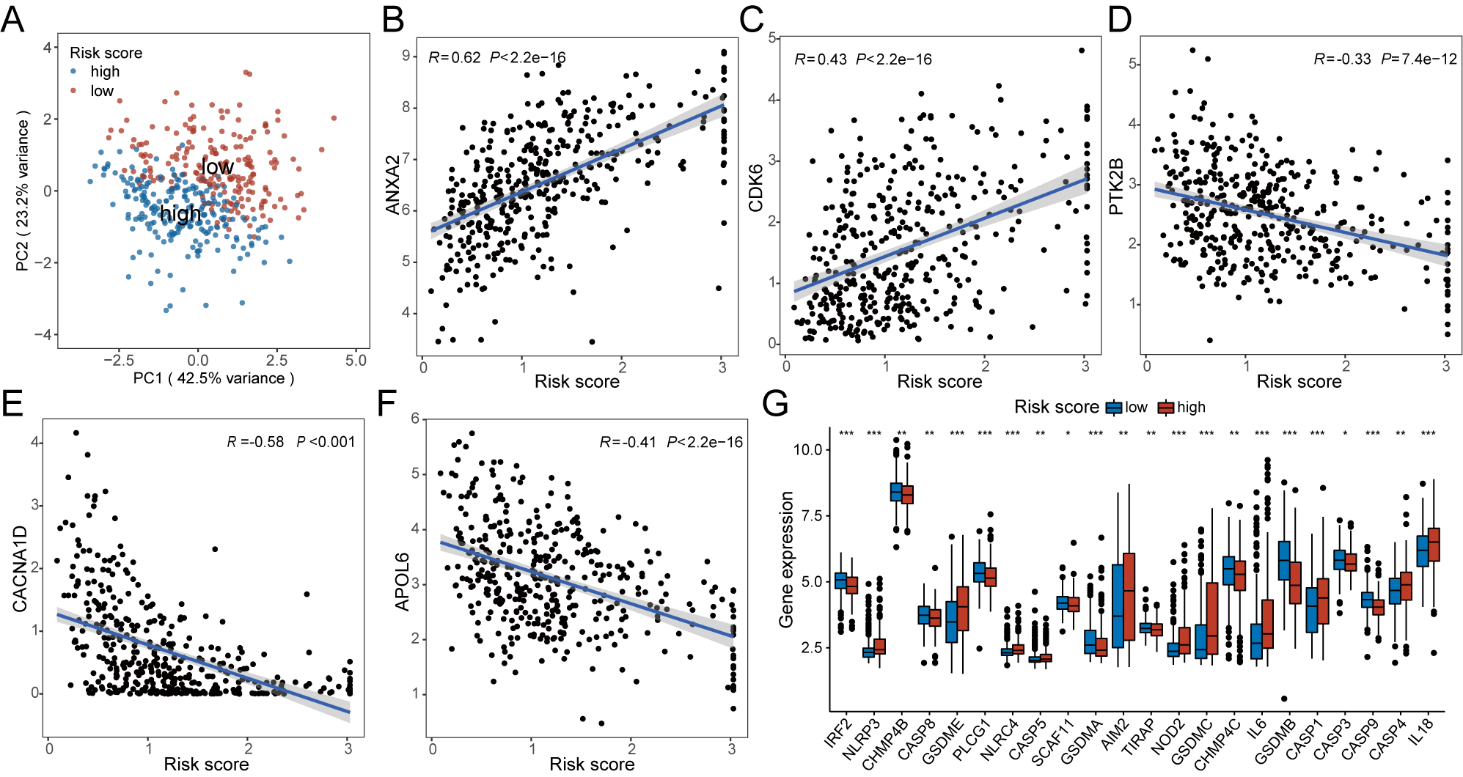


**Figure S2.** (A) PCA analysis showing a remarkable difference in transcriptomes between the two risk score groups. Correlations between risk score and ANXA2 (B), CDK6 (C), PTK2B (D), CACNA1D (E), APOL6 (F). (G) Expression of PRGs among high- and low-risk scores.


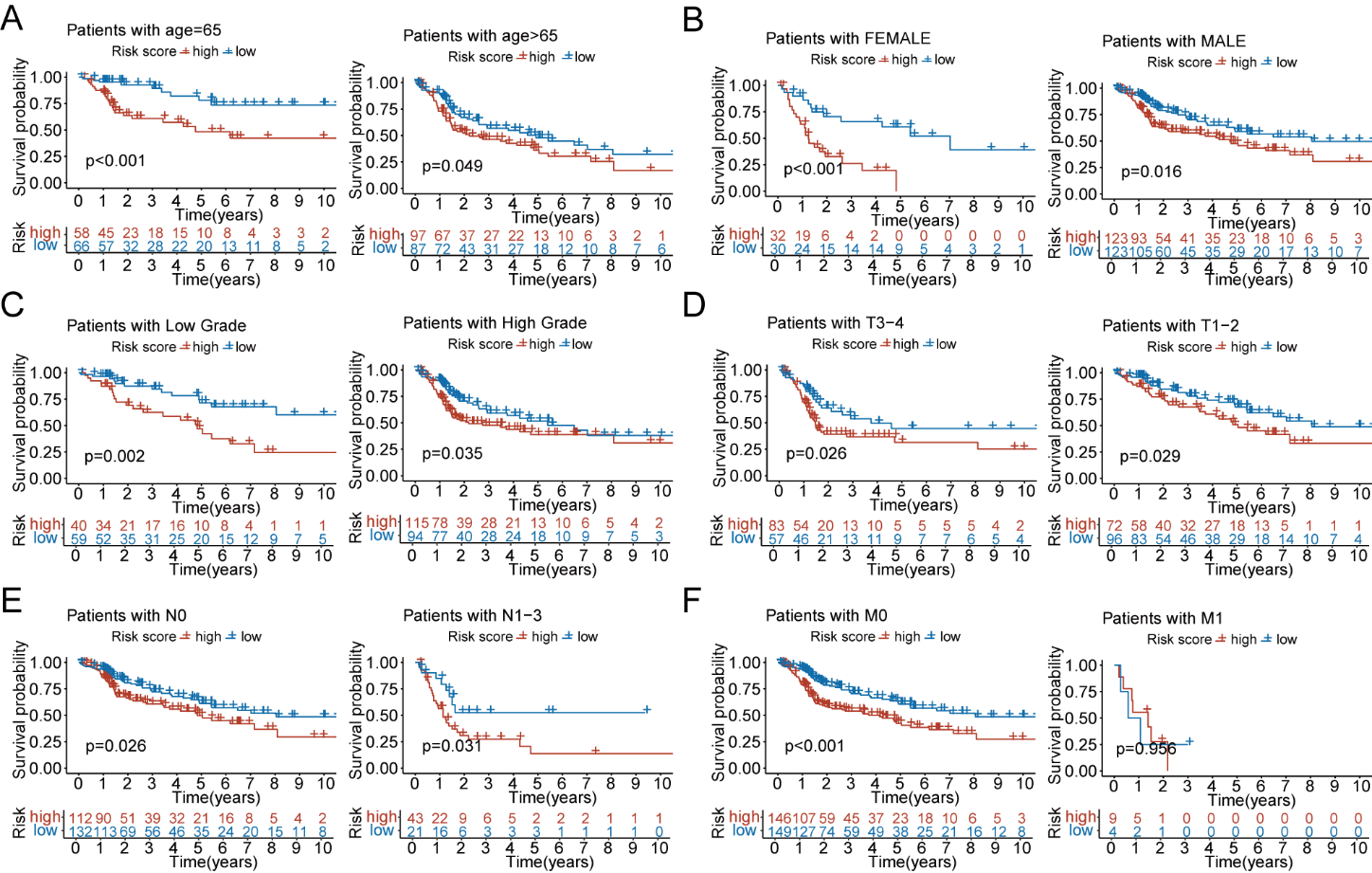


**Figure S3.** KM survival subgroup analysis of patients with BLCA according to the risk score stratified by clinical characteristics. (A) Age. (B) Gender. (C) Grade. (D) T stage. (E) N stage. (F) M stage.


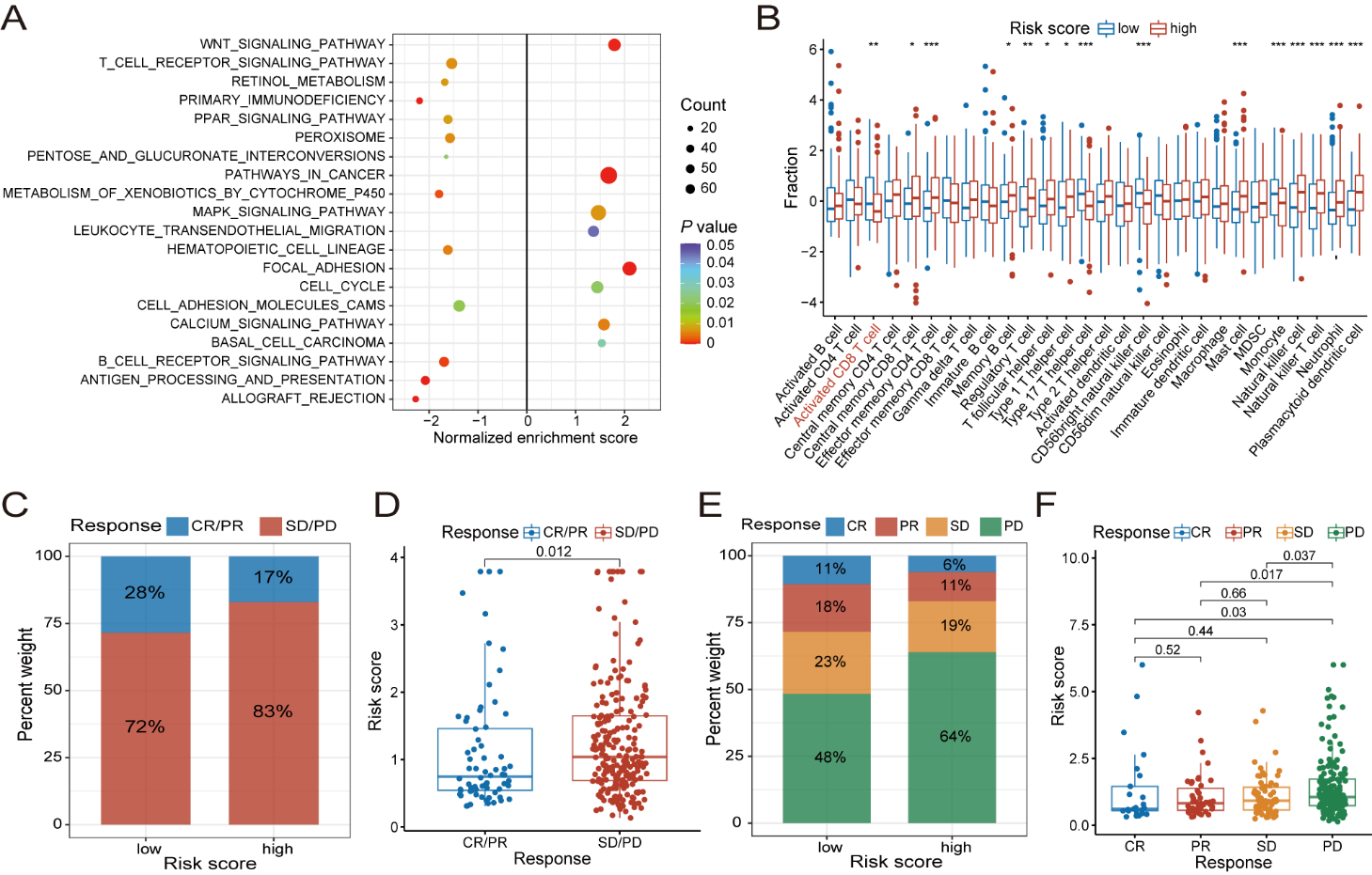


**Figure S4.** (A) Differences among the high- and low-risk scores groups in the state of signaling pathway. (B) Differences among the high- and low-risk scores groups in the abundance of infiltrating immune cells. (C) Proportions of anti-PD-L1 immunotherapy response (CR/PR vs. SD/PD) in high- and low-risk groups. (D) Differences of risk score in SD/PD and CR/PR groups. (E) Proportions of anti-PD-L1 immunotherapy response (CR, PR, SD, and PD) in high- and low-risk groups. (F) Differences of risk score in SD, PD, CR, and PR groups in IMvigor210 cohort.


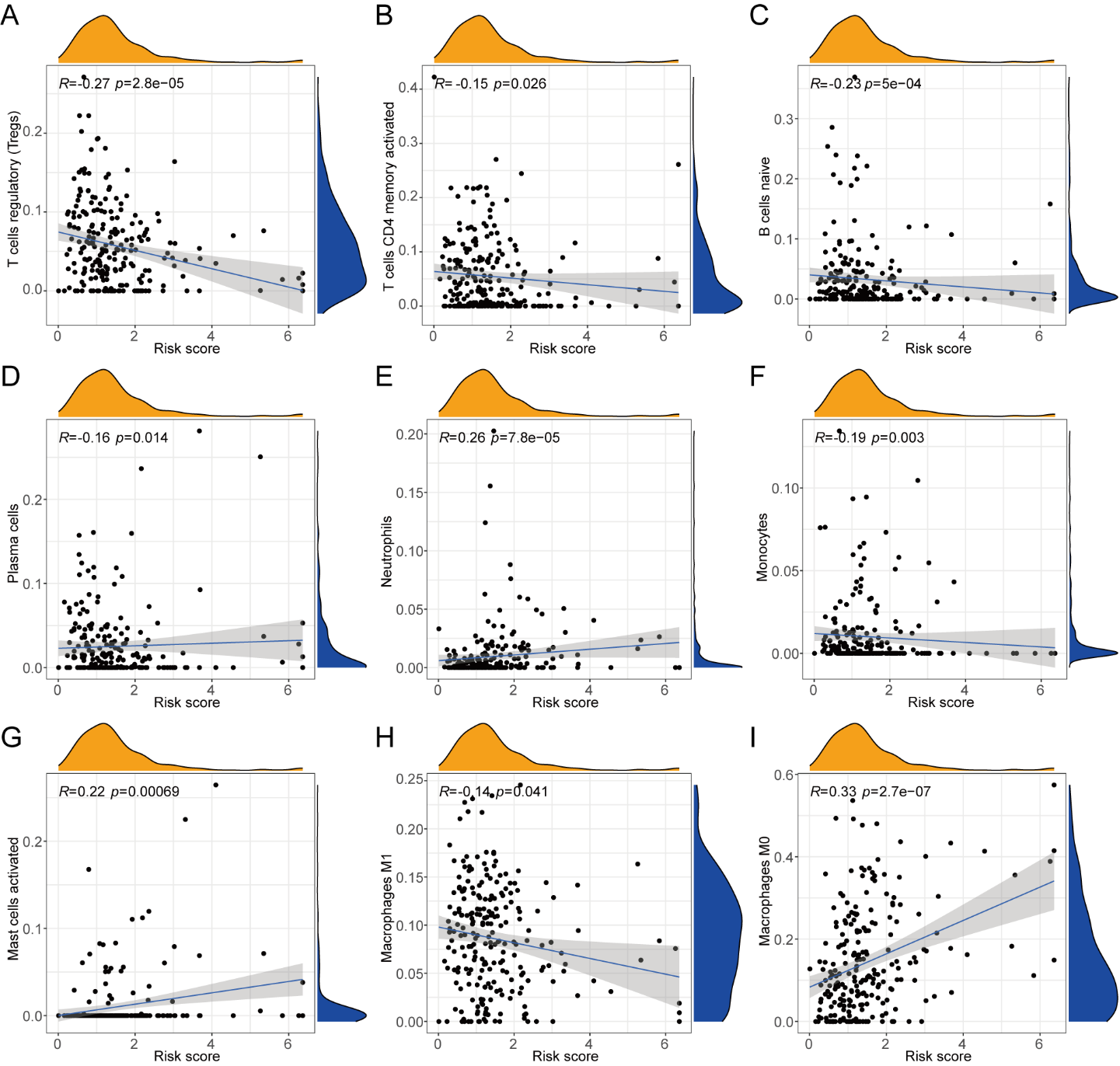


**Figure S5.** Correlations between risk score and immune cell types.

## Supplementary Tables

**Table S1.** Clinical information of BLCA patients.

| Variables | All patients (n = 577) |  | TCGA set (n = 412) |  | GEO set (n=165) |
| --- | --- | --- | --- | --- | --- |
|  | N (%) |  | N (%) |  | N (%) |
| **Age, y** |  |  |  |  |  |
| < 65 | 220 (38.13%) |  | 220 (36.65%) |  | 69 (41.82%) |
| ≥ 65 | 357 (61.87%) |  | 357 (63.35%) |  | 96 (58.18%) |
| **Sex** |  |  |  |  |  |
| Male | 439 (76.08%) |  | 439 (73.79%) |  | 135 (81.82%) |
| Female | 138 (23.92%) |  | 138 (26.21%) |  | 30 (18.18%) |
| **Grade** |  |  |  |  |  |
| Low Grade | 126 (21.84%) |  | 126 (5.10%) |  | 105 (63.64%) |
| High Grade | 448 (77.64%) |  | 448 (94.17%) |  | 60 (36.36%) |
| unknown | 3 (0.52%) |  | 3 (0.73%) |  | 0 (0%) |
| **T** |  |  |  |  |  |
| T1 | 83 (14.38%) |  | 83 (0.73%) |  | 80 (48.48%) |
| T2 | 151 (26.17%) |  | 151 (29.13%) |  | 31 (18.79%) |
| T3 | 215 (37.26%) |  | 215 (47.57%) |  | 19 (11.52%) |
| T4 | 70 (12.13%) |  | 70 (14.32%) |  | 11 (6.67%) |
| Unknown | 58 (10.05%) |  | 58 (8.25%) |  | 24 (14.55%) |
| **N** |  |  |  |  |  |
| N0 | 388 (67.24%) |  | 388 (58.01%) |  | 149 (90.30%) |
| N+ | 146 (25.30%) |  | 146 (31.80%) |  | 15 (9.09%) |
| Unknown | 43 (7.45%) |  | 43 (10.19%) |  | 1 (0.61%) |
| **M** |  |  |  |  |  |
| M0 | 354 (61.35%) |  | 354 (47.57%) |  | 158 (95.76%) |
| M1 | 18 (3.12%) |  | 18 (2.67%) |  | 7 (4.24%) |
| unknown | 205 (35.53%) |  | 205 (49.76%) |  | 0 (0%) |
| **Stage** |  |  |  |  |  |
| Stage I | 82 (14.21%) |  | 82 (0.49%) |  | 80 (48.48%) |
| Stage II | 157 (27.21%) |  | 157 (31.80%) |  | 26 (15.76%) |
| Stage III | 164 (28.42%) |  | 164 (34.22%) |  | 23 (13.94%) |
| Stage IV | 143 (24.78%) |  | 143 (33.01%) |  | 7 (4.24%) |
| unknown | 31 (5.37%) |  | 31 (0.49%) |  | 29 (17.58%) |

**Table S2.** Summary of 52 recognized pyroptosis-related genes.

| **Gene** | **Type** |
| --- | --- |
| BAK1 | pyroptosis |
| BAX | pyroptosis |
| CASP1 | pyroptosis |
| CASP3 | pyroptosis |
| CASP4 | pyroptosis |
| CASP5 | pyroptosis |
| CHMP2A | pyroptosis |
| CHMP2B | pyroptosis |
| CHMP3 | pyroptosis |
| CHMP4A | pyroptosis |
| CHMP4B | pyroptosis |
| CHMP4C | pyroptosis |
| CHMP6 | pyroptosis |
| CHMP7 | pyroptosis |
| CYCS | pyroptosis |
| ELANE | pyroptosis |
| GSDMD | pyroptosis |
| GSDME | pyroptosis |
| GZMB | pyroptosis |
| HMGB1 | pyroptosis |
| IL18 | pyroptosis |
| IL1A | pyroptosis |
| IL1B | pyroptosis |
| IRF1 | pyroptosis |
| IRF2 | pyroptosis |
| TP53 | pyroptosis |
| TP63 | pyroptosis |
| AIM2 | pyroptosis |
| CASP6 | pyroptosis |
| CASP8 | pyroptosis |
| CASP9 | pyroptosis |
| GPX4 | pyroptosis |
| GSDMA | pyroptosis |
| GSDMB | pyroptosis |
| GSDMC | pyroptosis |
| IL6 | pyroptosis |
| NLRC4 | pyroptosis |
| NLRP1 | pyroptosis |
| NLRP2 | pyroptosis |
| NLRP3 | pyroptosis |
| NLRP6 | pyroptosis |
| NLRP7 | pyroptosis |
| NOD1 | pyroptosis |
| NOD2 | pyroptosis |
| PJVK | pyroptosis |
| PLCG1 | pyroptosis |
| PRKACA | pyroptosis |
| PYCARD | pyroptosis |
| SCAF11 | pyroptosis |
| TIRAP | pyroptosis |
| TNF | pyroptosis |
| GZMA | pyroptosis |

**Table S3.** PCR primers of C1/C2 subtype marker genes and prognostic genes.

| **Gene** | **primer** |
| --- | --- |
| CXCL10 | Sense, 5′-GTGGCATTCAAGGAGTACCTC-3′ |
|  | antisense, 5′-TGATGGCCTTCGATTCTGGATT-3′ |
| CXCL9 | Sense, 5′-CCAGTAGTGAGAAAGGGTCGC-3′ |
|  | antisense, 5′-AGGGCTTGGGGCAAATTGTT-3′ |
| SPINK1 | Sense, 5′-TCTATCTGGTAACACTGGAGCTG-3′ |
|  | antisense, 5′-ACACGCATTCATTGGGATAAGT-3′ |
| DHRS2 | Sense, 5′-CCTCTGGTAGGGAGCACTCT-3′ |
|  | antisense, 5′-CCAGCGCCACTACTGGATTA-3′ |
| CACNA1D | Sense, 5′-CGCGAACGAGGCAAACTATG-3′ |
|  | antisense, 5′-TTGGAGCTATTCGGCTGAGAA-3′ |
| PTK2B | Sense, 5′-CCCCTGAGTCGAGTAAAGTTGG-3′ |
|  | antisense, 5′-GATACGCACGTCCTCCTTTTC-3′ |
| APOL6 | Sense, 5′-ACCAGGCGGAGAGAGAAAGT-3′ |
|  | antisense, 5′-TGTAGCTCCACGTCTTCACAC-3′ |
| CDK6 | Sense, 5′-TCTTCATTCACACCGAGTAGTGC-3′ |
|  | antisense, 5′-TGAGGTTAGAGCCATCTGGAAA-3′ |
| ANXA2 | Sense, 5′-TCTACTGTTCACGAAATCCTGTG-3′ |
|  | antisense, 5′-AGTATAGGCTTTGACAGACCCAT-3′ |
| GAPDH | Sense, 5′-CCTTCCGTGTCCCCACT-3′ |
|  | antisense, 5′-GCCTGCTTCACCACCTTC-3′ |

**Table S4.** The clinical features of BLCA patients.

| Characteristics | Sample A | Sample B |
| --- | --- | --- |
| Sex | Male | Male |
| Age | 84 | 69 |
| Pathology | High-grade papillary urothelial carcinoma | High-grade papillary urothelial carcinoma |
| T | T1 | T2 |
| N | N0 | N0 |
| M | M0 | M0 |
| Stage | I | II |
| Treatment | Transurethral resection of bladder tumor | Transurethral resection of bladder tumor |
